# Unraveling the Molecular Basis for Regenerative Cellular Plasticity

**DOI:** 10.1371/journal.pbio.0020232

**Published:** 2004-08-17

**Authors:** Shannon J Odelberg

## Abstract

Identifying the molecular basis for the impressive regenerative capacities of some organisms may help us to devise effective methods for enhancing regeneration in mammals

The regeneration of lost body parts and injured organs has captured the human imagination since the time of the ancient Greeks. The deep-seated roots of this early fascination can be seen in Greek mythology. The many-headed Hydra nearly defeated the hero Heracles by growing two new heads for every one that Heracles cut off, and the liver of Prometheus, devoured by a ravenous eagle each night, regenerated every morning. Aristotle, who lived from 384–322 BC, noted that the tails of lizards and snakes, as well as the eyes of swallow-chicks, could regenerate (Aristotle 1965). This fascination became a legitimate area of scientific inquiry in 1712, when the French scientist René-Antoine Ferchault de Réaumur published his seminal work on crayfish limb and claw regeneration (Réaumur 1712). Soon thereafter, several other prominent scientists of the eighteenth century, including Abraham Trembley, Charles Bonnet, Peter Simon Pallas, and Lazzaro Spallanzani, discovered remarkable regenerative abilities in a variety of organisms. Hydra, earthworms, and planarians could regenerate their heads and tails (Pallas 1766; Lenhoff and Lenhoff 1986); salamanders could regenerate their limbs, tails, and jaws; premetamorphic frogs and toads could regenerate their tails and legs; slugs could regenerate their horns; and snails could regenerate their heads (Spallanzani 1769). This last discovery caused quite a stir in eighteenth-century France, leading to an “unprecedented assault” on snails as both naturalists and the general public participated in the quest for scientific knowledge by reproducing Spallanzani's intriguing results (Newth 1958).

## Stem Cells Versus Dedifferentiation

**Figure 1 pbio-0020232-g001:**
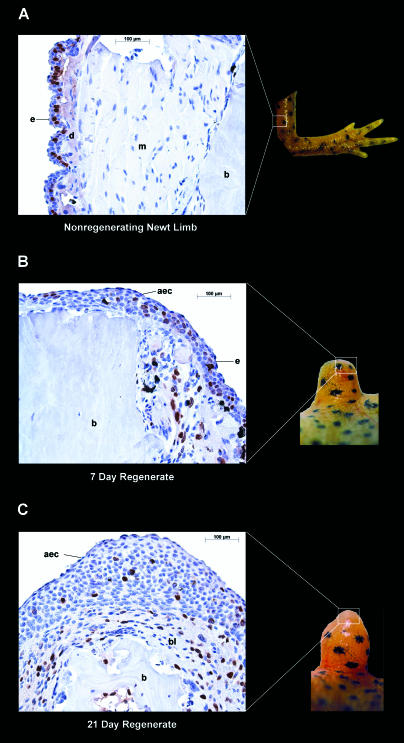
Dedifferentiation of Limb Cells During Salamander Limb Regeneration Brown nuclei are a result of BrdU incorporation during DNA synthesis, and therefore mark cells that are progressing through the cell cycle. Abbreviations: e, epidermis; d, dermis; m, muscle; b, bone; bl, blastema; aec, apical epithelial cap. (A) Unamputated right forelimb of a newt and coronal section of the stylopodium. The only cells actively synthesizing DNA are those in the basal layer of the epidermis (bone marrow cells also actively synthesize DNA in the unamputated limbs but are not shown here). Note the long myofibers in the nonregenerating newt limb and the distant spacing between the muscle nuclei. (B) Seven-day limb regenerate and coronal section of the distal regenerating tip. Note that the muscle cells have lost their normal architecture and that the nuclei are more closely spaced and have begun to synthesize DNA. (C) Twenty-one-day limb regenerate and coronal section of the distal regenerating tip. The nuclei of the blastema are spaced closely together, and many nuclei are actively synthesizing DNA. The bone is also being broken down in the vicinity of the blastema.

During the nineteenth century and for most of the twentieth century, regeneration research primarily focused on the phenomenology of regeneration and its cellular basis. Many important discoveries were made during this period, which led in part to the general conclusion that progenitor cells are required for most regenerative processes. However, the origin of these progenitor cells varies between regenerating systems. In some cases, such as the regeneration of skin, blood, muscle, and bone in mammals and the replacement of lost tissues in the flatworm planarian, the progenitor cells pre-exist as reserve cells or stem cells that only need to be activated in response to injury or tissue depletion. In other cases, the progenitor cells can be created de novo through a process in which fully differentiated cells reverse their normal developmental processes and revert to proliferating progenitor cells. This latter process, known as cellular dedifferentiation, is especially prominent in vertebrates with exceptional regenerative abilities, such as salamanders. For example, during salamander limb regeneration, cells from muscle, bone, cartilage, nerve sheath, and connective tissues participate in the dedifferentiation process to form a pool of proliferating progenitor cells known as the regeneration blastema (Figure 1) (Chalkley 1954; Bodemer and Everett 1959; Hay and Fischman 1961; Wallace et al. 1974; Lo et al. 1993; Kumar et al. 2000). It has not yet been determined whether pre-existing stem cells or reserve cells also contribute to the pool of progenitor cells—nor whether the blastemal cells are multipotent (capable of differentiating into multiple cell types), are committed to a particular cell lineage, or are a mix of multipotent and committed progenitor cells. Regardless, these blastemal cells will later redifferentiate to form all the internal tissues of the regenerated limb other than the peripheral nerve axons. This extraordinary degree of cellular plasticity distinguishes those vertebrates that can replace entire anatomical structures, such as limbs, from vertebrates with more limited regenerative abilities.[Fig pbio-0020232-g001]

The public has recently exhibited a renewed interest in regeneration research, due in large part to stem cell research, which has provided promising avenues for the field of regenerative medicine. In addition, celebrities such as Christopher Reeve and Michael J. Fox have given a human face to the many people who could benefit from effective regenerative therapies. The political, ethical, and religious controversies surrounding the use of human embryonic stem cells for therapeutic purposes have only served to increase the public's awareness of the promising potential of regenerative medicine. But this interest in using scientific knowledge to enhance the regenerative capacity in humans is not new. Spallanzani closed his 1768 monograph on regeneration, *An Essay on Animal Reproductions*, with a series of questions— which, except for the antiquated language, could be asked by citizens of the twenty-first century: But if the abovementioned animals, either aquatic or amphibious, recover their legs, even when kept on dry ground, how comes it to pass, that other land animals, at least such as are commonly accounted perfect, and are better known to us, are not endued with the same power? Is it to be hoped they may acquire them by some useful dispositions? [A]nd should the flattering expectation of obtaining this advantage for ourselves be considered entirely as chimerical?

Although most of the current interest in regenerative medicine focuses on the potential benefits of either embryonic or adult stem cells, there are several investigators who are now taking an entirely different approach to the problem. These researchers think that although stem cells may offer some benefits in the relatively near future, a more comprehensive approach will be required to meet all of our regenerative needs. To achieve this goal, they must first learn how nature has already solved the problem of regeneration and then use this information to enhance the regenerative capacity in mammals. These studies seek to understand the biology of regeneration, especially the cellular and molecular mechanisms that govern regenerative processes. The experimental systems range from the unicellular protozoa to complex vertebrates, such as salamanders and mice.

## The Molecular Biology of Regeneration

With the technological advances that followed the advent of molecular biology, researchers acquired the basic tools to begin to unravel the molecular basis for cellular plasticity and regeneration. However, progress in this arena has been slow, given that most organisms with marked regenerative abilities are not yet amenable to routine genetic manipulation. Recent advances, such as the application of mutagenic screens to study fin regeneration in zebrafish (Johnson and Weston 1995; Poss et al. 2002b) and the application of RNAi knockdown technology to study regeneration in planarians (Sanchez Alvarado and Newmark 1999; Newmark et al. 2003), are quite promising and could largely ameliorate this deficiency. Nevertheless, results from several recent studies have converged on a set of genes that appear to play an important role in regeneration, and evidence is accumulating that suggests some of these genes may function to control regenerative cellular plasticity. Three such genes are *Msx1, BMP4*, and *Notch1*. These genes encode, respectively, a transcriptional repressor, a signaling ligand, and a signaling cell surface receptor.

Numerous studies over the past three decades have shown that mammals, including humans, can regenerate their digit tips provided the amputation plane is distal to the terminal phalangeal joint (Douglas 1972; Illingworth 1974; Borgens 1982; Singer et al. 1987). However, *Msx1*-deficient mice exhibit impaired fetal digit-tip regeneration, a phenotype that can be rescued in ex vivo cultures in a dose-dependent manner by application of exogenous BMP4 (Han et al. 2003). Recently, it has been demonstrated that Xenopus tadpoles are unable to regenerate their tails during a refractory period of development between stages 45 and 47 (Beck et al. 2003). If tails are amputated during this refractory period, genes that are normally expressed during the early stages of tadpole tail regeneration, such as *BMP4, Msx1*, and *Notch-1*, are not expressed. However, transgenic frogs carrying a hyperactive form of *Msx1* or constitutively active *ALK3* (a receptor for BMP4) are able to regenerate their tails during the refractory period. Transgenic frogs carrying a constitutively active *Notch-1* receptor will regenerate their notochords and spinal cords but exhibit little or no muscle regeneration, suggesting that Notch-1 signaling alone cannot rescue complete regenerative capacity in frog tadpoles (Beck et al. 2003). Results from expression studies in a variety of organisms are consistent with these in vivo gene function studies. *Msx* genes are upregulated in regenerating salamander limbs and regenerating zebrafish fins and hearts (Simon et al. 1995; Poss et al. 2002a; Raya et al. 2003), while *notch-1b* and its ligand, *deltaC*, are upregulated during zebrafish heart and fin regeneration (Raya et al. 2003).

## *Msx1* and Cellular Plasticity

Although these functional and expression studies indicate that *Msx1, Bmp4*, and *Notch-1* are important for a variety of regenerative processes, they do not address the mechanism by which these genes exert their effects. However, several in vitro studies suggest that *Msx1* may be involved in regulating cellular plasticity. Ectopic expression of *Msx1* can inhibit the differentiation of a variety of mesenchymal and epithelial progenitor cell types (Song et al. 1992; Hu et al. 2001), suggesting that this gene may play a role in maintaining cells in an undifferentiated state. Furthermore, *Msx1* may be functioning not only to prevent differentiation of progenitor cells but also to induce dedifferentiation of cells that have already differentiated. Ectopic expression of *Msx1* in mouse myotubes (differentiated muscle cells that are multinucleated and are able to contract), coupled with serum stimulation, can induce these multinucleated cells to reduce their levels of myogenic proteins and undergo a cell cleavage process that produces proliferating mononucleated cells (a process known as cellularization) (Odelberg et al. 2000). Clonal populations of these dedifferentiated cells can redifferentiate into cells expressing markers for cartilage, fat, and bone cells, as well as myotubes. These results suggest that the combination of ectopic *Msx1* expression and serum stimulation can induce differentiated muscle cells to dedifferentiate into proliferating multipotent progenitor cells. Given this degree of cellular plasticity, it is not surprising that *Msx1* can also induce muscle progenitor cells, known as myoblasts, to dedifferentiate to multipotent progenitor cells (Odelberg et al. 2000).

Cellularization of myotubes and myoblast dedifferentiation can also be induced by at least two synthetic trisubstituted purines. Myoseverin is a trisubstituted purine that binds to and disassembles microtubules, leading to the cellularization of multinucleated myotubes (Rosania et al. 2000). The resulting mononucleated cells proliferate when stimulated with serum and redifferentiate into myotubes following serum starvation. A second trisubstituted purine, reversine, induces myoblasts to dedifferentiate into progenitor cells with adipogenic and osteogenic potential (Chen et al. 2004). Therefore, reversine and *Msx1* appear to have a similar effect on mouse myoblasts, although no reports have yet addressed whether reversine might induce dedifferentiation of multinucleated myotubes.

In this issue of *PLoS Biology*, Kumar et al. (2004) present data linking *Msx1* function to microtubule disassembly during the process of salamander myofiber cellularization and fragmentation (myofibers are formed from myotubes and represent the completely mature form of the differentiated skeletal muscle cell). Their results suggest that *Msx1* expression induces microtubule disassembly, which then leads to myofiber cellularization or fragmentation. If *Msx1* function is markedly reduced in salamander myofibers by preventing the efficient synthesis of the Msx1 protein, cellularization or fragmentation of the myofiber is inhibited, suggesting that Msx1 is required for this process. Thus, this study complements previous work (Odelberg et al. 2000) showing that ectopic *Msx1* expression, coupled with serum stimulation, is sufficient to induce cleavage, cellularization, and dedifferentiation of mouse myotubes. The two studies point to an essential role for *Msx1* in regenerative cellular plasticity and when combined with previous in vivo studies, raise the possibility that BMP or Notch signaling might also play a role in this process.

Results from these and other similar studies are beginning to give researchers a glimpse into the molecular mechanisms that control regeneration and cellular plasticity. With the new tools available to identify candidate genes and assess their function, the next few decades appear promising for scientists engaged in regeneration research. Elucidating the molecular basis for regeneration may prove to be an essential step in devising effective methods for enhancing regeneration in mammals and may well usher in a golden era for regenerative medicine.

## Accession Numbers

The Mouse Genome Informatics (http://www.informatics.jax.org/) accession numbers of the genes discussed in this paper are *ALK3* (MGI: 1338938), *BMP4* (MGI: 88180), *Msx1* (MGI: 97168), and *Notch1* (MGI: 97363). The GenBank (http://www.ncbi.nih.gov/GenBank/) accession numbers of the genes discussed in this paper are *deltaC* (NM 130944), *notch1b* (Y10352), Ambystoma mexicanum *Msx1* (AY525844), Danio rerio msxb (U16311; partial sequence), D. rerio msxc (NM 131272), Homo sapiens *ALK3* (Z22535), and Mus musculus *ALK3* (Z23154).
